# Combination of EZH2 inhibitor and BET inhibitor for treatment of diffuse intrinsic pontine glioma

**DOI:** 10.1186/s13578-017-0184-0

**Published:** 2017-10-30

**Authors:** Yaqin Zhang, Weijie Dong, Junying Zhu, Lizhu Wang, Xinjian Wu, Hong Shan

**Affiliations:** 1 Department of Radiology, The 5th Affiliated Hospital of Sun Yat-sen University, No. 52 Meihua Dong Road, Zhuhai, 519000 Guangdong Province People’s Republic of China; 2 Neurosurgery Department, The 1st Affiliated Hospital of Sun Yat-sen University, No. 58 Zhongshan No. 2 Road, Guangzhou, 510030 Guangdong Province People’s Republic of China; 3 Department of Radiology, The 3rd Affiliated Hospital of Sun Yat-sen University, No. 600 Tianhe Road, Guangzhou, 510630 Guangdong Province People’s Republic of China; 4 Department of Interventional Medicine, The 5th Affiliated Hospital of Sun Yat-sen University, No. 52 Meihua Dong Road, Zhuhai, 519000 Guangdong Province People’s Republic of China

**Keywords:** DIPG, EZH2 inhibitor, BET inhibitor, Epigenetics, Tumor therapy

## Abstract

**Background:**

Diffuse intrinsic pontine glioma is an infiltrative, often high-grade glioma of the brainstem that is not amenable to surgical resection. The current treatment of DIPG by radiation therapy showed dramatically improvement of patient’s condition, however, the tumor recurs rapidly. More and more studies are focused on the genetic and epigenetic drivers of DIPGs, which may provide more and more novel therapy target for DIPG. EZH2 has been proved to be a potential therapeutic target for H3K27M-mutant pediatric gliomas recently. Meanwhile, BET family protein is a hot target in many different types of cancers, including DIPG. In this study, we performed the treatment of both EZH2 and BET inhibitor for DIPG cells.

**Results:**

The combination of these two inhibitors exhibited better inhibition of the tumor growth both in vitro and in vivo compared to use the inhibitor individually. This inhibition was performed by blocking the proliferation and promoting the cell apoptosis. Meanwhile, combination treatment of these two inhibitors would also affect the epigenetic markers which were abnormal in the tumors of the certain set of genes.

**Conclusion:**

Thus we provided a novel therapy strategy for clinical treatment of DIPG.

## Background

Diffuse intrinsic pontine glioma (DIPG), a tumor located in the middle of the brain stem, is a fatal malignant pediatric brain tumor, which is the leading cause of cancer‑related mortality in children [1]. The 5-year survival rate of DIPG is < 1%. The median overall survival of children diagnosed with DIPG is approximately 9 months and the 1- and 2-year survival rates are approximately 30% and less than 10%, respectively [2]. So far, radiotherapy, which offers a significant but transient improvement, is the standard treatment of DIPG, while chemotherapy has not shown any benefit [1, 3]. So more understanding of the molecular mechanism of DIPG is required to find the new target and develop more specifically therapeutic approaches for DIPG.

Nearly 80% of DIPGs harbor histone H3 mutations, wherein lysine 27 is substituted with methionine (H3K27M) [4–8]. DIPGs expressing H3K27M mutant will reduce the global levels of H3K27me3 [9], which is mediated by PRC2 [10]. Polycomb repressive complexes (PRCs), including PRC1 and PRC2, mediate gene silencing by posttranslational modification of histones [11, 12]. The PRC2 complex takes responsibility for trimethylation of Lys 27 of histone H3 (H3K27me3), this modification is catalyzed by its enzymatic subunits EZH1 and EZH2 [13]. EZH2 is actively involved in many cellular processes such as cell cycle progression, cell proliferation, cell differentiation and apoptosis [14]. EZH2 mutations have been found to relate to multiple human cancers [15]. Recent study shows that EZH2 activity is required for the growth of mouse DIPG tumor cells in vitro [16].

The bromodomain and extraterminal (BET) family proteins, which playing a key role as epigenetic regulators, are responsible for the transcriptional activation by interaction with acetylated [17, 18] chromatin [19, 20]. BET proteins regulate the expression of many important oncogenes, which involved in apoptosis and cell cycle arresting [21–23]. Therefore, small molecule inhibitors of BET proteins have been developed and proved to be active in both solid and hematologic malignancies, including brain tumors [24, 25]. JQ1, reported by Filippakopoulos et al. is a small molecule that competitively binds to bromodomains with high potency and specificity. Taylor et al. found that combination targeting MYCN and NOTCH by JQ1 and MRK003 inhibited DIPG growth and induced apoptosis, suggesting this may work as an effective therapeutic strategy in DIPG.

In this study, we performed the treatment of both EZH2 and BET inhibitor for DIPG cells in order to examine whether combination treatment would be better than the treatment of the inhibitor individually. This study was aim to find the new strategy of chemotherapy for the treatment of DIPG.

## Methods and materials

### Cell lines and culture

NSCs were isolated from the dorsal forebrain of mouse embryos at E12.5. After the embryos were isolated, the skin is removed, then the dorsal forebrains were dissected out and incubated in 0.25% trypsin–EDTA (GIBCO, Grand Island, NY, USA) at 37 °C for 20 min. The tissue was dissociated by pipette thoroughly, and then cultured in the poly-d-lysine (PDL, Sigma-Aldrich, St. Louis, MO, USA)- and laminin (Sigma-Aldrich, USA)-coated plates in neural stem cell medium. The neural stem cell medium contained 50% DMEM-F12, 50% neurobasal medium, N2 and B27 supplements, sodium pyruvate, glutamax, HEPES, β-mercaptoethanol, non-essential amino acids, bovine serum albumin, heparin, 100 U/ml penicillin, 100 μg/ml streptomycin, human recombinant epidermal and basic fibroblast growth factors. After 3 days culture, the cells were treated with 0.25% trypsin–EDTA and snap freezed by liquid nitrogen in NSC medium supplemented with 10% DMSO.

### Reagents

DMEM-F12 was bought from GIBCO (USA). The charcoal-stripped fetal calf serum (FCS) was purchased from HyClone Laboratories (Inc., Logan, UT, USA). Anti-mouse Flag-tag, HA-tag, H3, H3K27me3 and GAPDH were bought from Sigma-Aldrich (Inc, USA). Cell counting kit-8 was bought from Dojindo Laboratories (Inc, Japan). The EZH2 inhibitor EPZ6438 and BET inhibitor JQ-1 were from Selleck Chemicals.

### Flow cytometry and apoptosis

Cells with different treatments were washed twice in FACS medium phosphate buffered PBS containing 1% FCS and 0.1% NaN_3_. Then the cells were washed by AnnexinV binding buffer for 3 times. After centrifugation and discuss the supernatant, cells were incubated for 30 min at 4 °C with FITC-AnnexinV according to the standard procedure. PI was added before testing. Fluorescence was measured by using a FACSCalibur (Becton–Dickinson, San Diego, CA) and data were analyzed by using the Flowjo Software (Becton–Dickinson, San Diego, CA).

### Chromatin immunoprecipitation (ChIP)

Around 5 × 10^7^ cells were incubated with 37% Formaldehyde diluted to a 1% final concentration for crosslinking for 15 min at room temperature. 1 M Glycine diluted to a final concentration of 125 mM was added to stop the crosslink at room temperature for 5 min. Cells were pelleted and resuspent in 5 ml of Lysis Buffer (10 μg/ml Leupeptin, 10 μg/ml Aprotinin, and 1 mM PMSF) and aliquoted to the 1.5 mL eppendorf tubes 500 μl each, then the samples were incubated on ice for 10 min. Samples were sonicated by Bioruptor™ UCD-200 to generate 500 bp–1 kb length DNAs. Then 500 μl of each sample was centrifuged for 10 min at 12,000*g*. Supernatant was collected and diluted by adding 1 ml of Dilution Buffer (containing the same amount of protease inhibitors as in Lysine Buffer) and add 5 μg of the antibody or normal IgG to the samples. Tubes were incubated at room temperature for 15 min and then secondary antibody was added into the tubes, after another incubation at room temperature for 15 min, 4 times of washing were performed by Wash Buffers pre-chilled to 2 to 8 °C. After the final wash and centrifugation, 120 μl of deionized or distilled water was added to the system to resuspend the DNAs. 2–10 μl of the DNA sample was used in the q-PCR reactions.

### Soft-agar colony formation assay

Cells with different treatments were harvested and pipetted well to become single-cell suspension in complete culture media in a concentration of 1 × 10^6^/ml. Then the cells were incubated at room temperature for using. 10% FBS DMEM was pre-warmed at 37 °C and 4% agar was melted by microwave and keep warm in 56 °C water bath. 0.9 ml 4% agar and 4.1 ml pre-warmed of 10% FBS DMEM were mixed well and put into 60-mm culture dish in the hood. After it became solid, 3 × 10^4^ cells, 2.73 ml pre-warmed 10% FBS DMEM and 270 μl of 4% pre-warmed agar were mixed together to form the top gel. After the gel became solid, the dish were incubated at 37 °C for 3 weeks. Then the colonies were stained with 0.04% crystal violet-2% ethanol in PBS.

### Western blot analysis

Cells (1 × 10^7^) were lysed in a buffer containing 20 mM Tris–HCl (pH 7.6), 250 mM NaCl, 0.5% NP-40, 3 mM EDTA and 1.5 mM EGTA with 10 μg/ml Aprotinin, 10 μg/ml leupeptin, 1 mM DTT, 1 mM PNPP and 0.1 mM Na_3_VO_4_ as protease and phosphatase inhibitor. After centrifugation, cell lysates (100 μg/lane) were subjected to 10% SDS-PAGE and transferred onto polyvinylidene difluoride membranes (Roche, Germany). The membranes were blocked for 1 h in TBST (25 mM Tris–HCl, pH 7.6, 125 mM NaCl, 0.1% Tween-20) containing 5% nonfat dried milk, and then the membrane was incubated with antibodies against Flag-tag, HA-tag, H3K27me3, H3 or GAPDH was diluted in TBST containing 5% nonfat dried milk at 4 °C overnight. HRP conjugated goat anti-rabbit or anti-mouse antibodies were used as second antibodies. All the antibodies were from Sigma. Protein bands were detected by the Immobilon Western Chemiluminescent HRP Substrate (Millipore, Billerica, MA, USA) and images were taken by FluorChem FC2 System (Alpha Innotech Corporation, USA).

### The cell proliferation assay

The cell proliferation was detected cell counting or by a Cell Counting Kit-8 according to the manufacturer’s instructions.

### Animals and surgical procedures

Six-week-old female BALB/c mice were provided by the animal center in Sun Yat-sen University. All protocols, described below, were approved by the Animal Care and Use Committee of Sun Yat-sen University. 1 × 10^5^ PDGFB/H3K27wt or PDGFB/H3K27M cells suspended in 1 μl Hank’s balanced salt solution without Ca^2+^ and Mg^2+^ were injected slowly (over 1 min) into the pontine tegmentum at 5-mm depth from the inner base of the skull. Inhibitors were injected by intraperitoneal (i.p.) injection with the amount as indicated in the figure legend 5 days after the tumor generation. Mice were monitored daily and recorded the survival rate.

### Statistical analysis

Results were expressed as mean ± SD. p values were determined using two-tailed Student’s *t* test. p values were indicated in each figure.

## Results

### H3K27M is sufficient to generate tumors

**Fig. 1:**
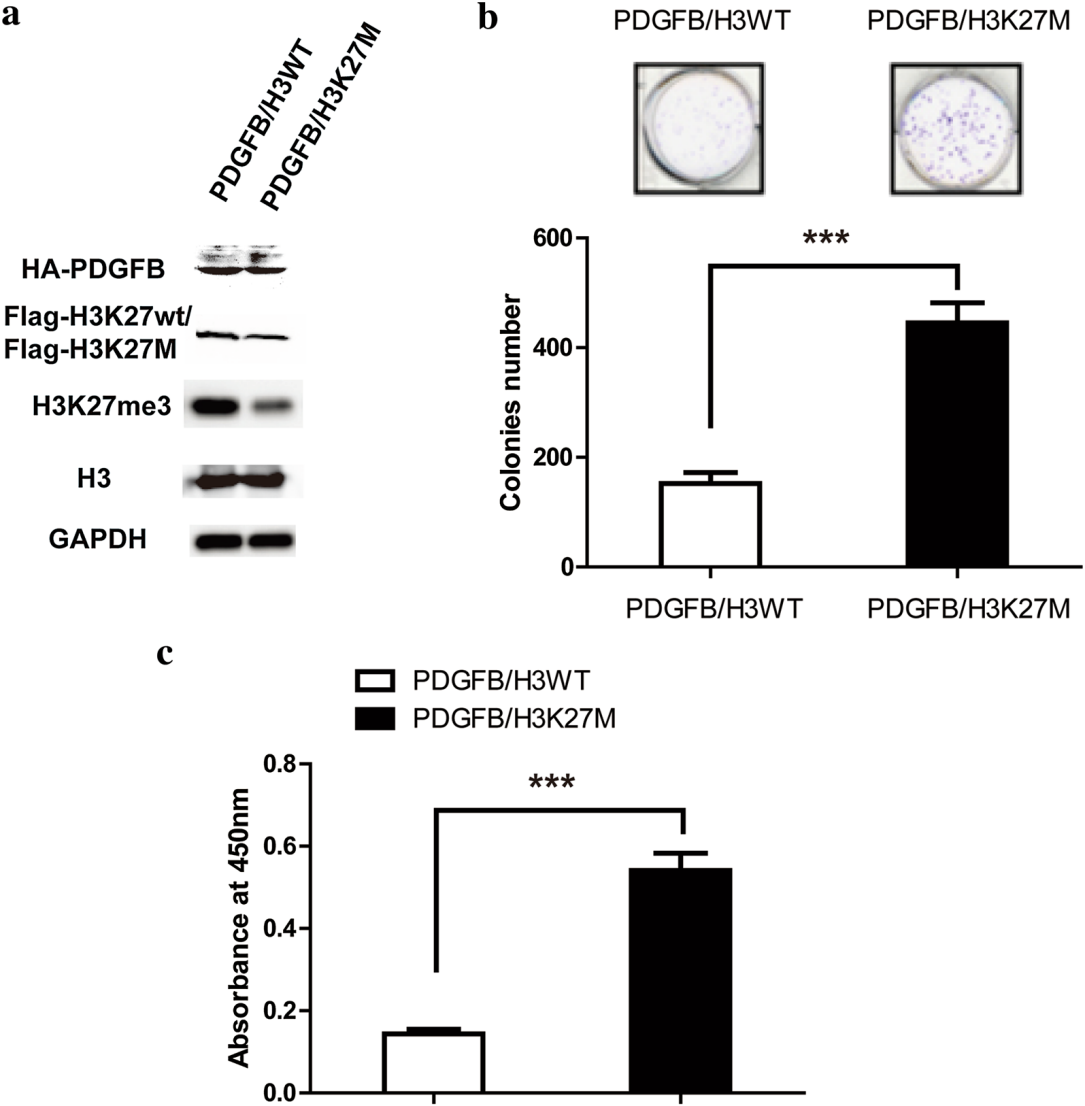
H3K27M make the NSCs gain the tumor activity. **a** Western blot showing the expression level of Flag-tagged-H3K27M or Flag-tagged-H3K27wt, HA-tagged-PDGFB, H3K27me3, H3 and GAPDH. **b** Soft agar colony assay of PDFGB/H3WT or PDGFB/H3K27M NSCs. Data were represented as mean ± SD, n = 3 independent experiments. ***p < 0.001. **c** Cck-8 kit was used to evaluate the viability of PDFGB/H3WT or PDGFB/H3K27M NSCs. Data were represented as mean ± SD, n = 3 independent experiments. ***p < 0.001

**Fig. 2:**
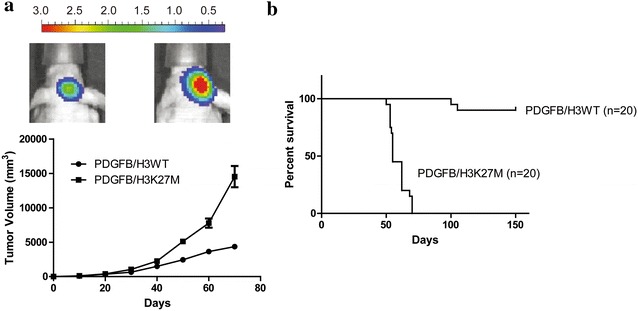
H3K27M is sufficient to generate DIPG tumors. **a** Tumor generated by PDFGB/H3WT or PDGFB/H3K27M NSCs was measured in diameters and calculated to volume according to the time point. **b** Survival curve of the mice injected into the pons with PDFGB/H3WT or PDGFB/H3K27M NSCs (1 × 10^5^). Each group contains 20 mice

As reported, H3K27M-mutant expression in DIPGs is associated with up-regulation of PDGF signaling [4, 5], so we expressed Flag-tagged K27M H3.3 mutant or Flag-tagged H3K27wt in mouse neural stem cells (NSCs) expressing HA-tagged-PDGFB. Figure 1a showed the NSC cells expressed with both Flag-tagged H3K27M and HA-tagged-PDGFB exhibited a global reduction of H3K27me3. Consistent with other’s reports [4, 9, 16, 26], these PDGFB/H3K27M NSCs had an improved ability of colony-forming compared to the PDGFB/H3K27wt cells (Fig. 1b). Meanwhile, the PDGFB/H3K27M NSCs grew faster than the PDGFB/H3K27wt cells, which made the PDGFB/H3K27M NSCs could generate the larger tumor when implanted to the pons of the mice (Fig. 1c). Then we used mice model to evaluate the tumor formation ability of the modified NSCs. The mice implanted the PDGFB/H3K27M NSCs developed larger tumors than the wt cells (Fig. 2a), and PDGFB/H3K27M group had poor survival rate compare to the PDGFB/H3K27wt (Fig. 2b). These results were consistent with the recent research and indicated that H3K27M is sufficient to generate DIPG.

### Combination of EZH2 and BET inhibitors on the tumor cells proliferation and apoptosis

**Fig. 3:**
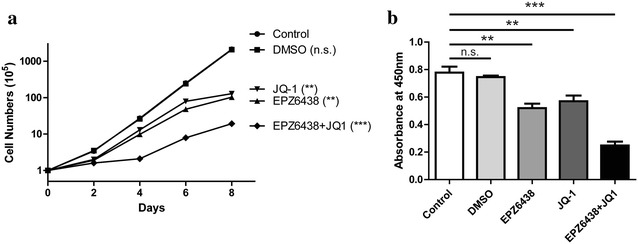
Combination of EZH2 and BET inhibitors reduced the cell proliferation in DIPG cells. **a** Cell proliferation of different groups as indicated was determined by cell counting. The concentration of EPZ6438 was 3 μM and JQ-1 was 300 nM, the following in vitro assay used the same amount of the inhibitors. **p < 0.01 and ***p < 0.001. **b** Cck-8 kit was used to evaluate the viability of each group of the cells as indicated. Data were represented as mean ± SD; n = 3 independent experiments. **p < 0.01 and ***p < 0.001

**Fig. 4:**
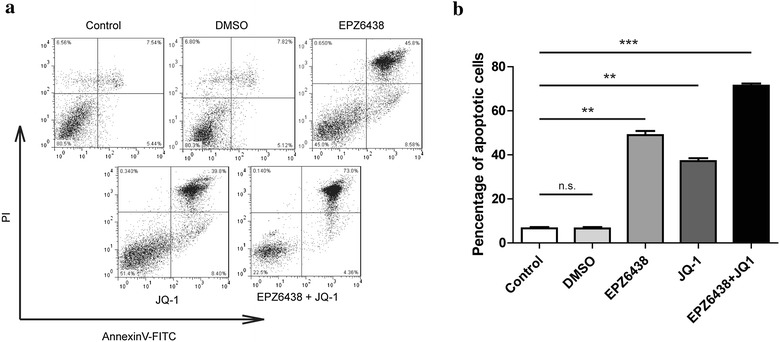
Combination of EZH2 and BET inhibitors promoted the cell apoptosis in DIPG cells. **a** The apoptosis of the DIPG cells with different treatment as indicated was determined by using the Annexin-V-FITC & PI Apoptosis Kit and assessed by flow cytometry. n = 3 independent experiments and this panel presented one of these repeats. **b** Statistic of percentage of the apoptosis cells performed in **a**. Data showed the Annexin-V and PI double positive cells. Data were represented as mean ± SD; n = 3 independent experiments. **p < 0.01 and ***p < 0.001

Although H3K27M mutant tumor cells exhibited the global reduction in H3K27me3 levels (Fig. 1a), recent studies showed that several genes, especially several tumor-suppressor genes, retained or even showed increased H3K27me3 levels [27, 28]. Due to this point, EZH2 activity has been proved to be required for the growth of mouse DIPG cells in vitro and in vivo [16]. In the other hand, BRD2 and BRD4 proteins were found to co-occupy with H3K27M-K27ac, then logically, the BET inhibitor was also demonstrated could efficiently inhibit tumor progression [29]. Therefore, we thought these two inhibitor may both be potential for clinical trial, so we tested the effect of combination of these two inhibitors. The cell counting (Fig. 3a) and cell viability presented by cck-8 kit (Fig. 3b) indicated that both of EZH2 inhibitor (EPZ6438) and BET inhibitor (JQ-1) could reduce the proliferation of PDGFB/H3K27M NSCs. Interestingly, combination of these two inhibitors exhibited better reduction compare to only using one inhibitor (Fig. 3a, b). We got the similar results on the apoptosis assay by FITC-AnnexinV and PI staining. The PDGFB/H3K27M NSCs showed the very low basal apoptotic ratio (Fig. 4a). Treatment of EPZ6438 or JQ-1 remarkably promoted the apoptosis and the combination of these two inhibitors showed the further induction of apoptosis (Fig. 4a, b). Thus, we have demonstrated that EZH2 and BET proteins activity were required for the growth of PDGFB/H3K27M NSCs, inhibition of these two group of proteins showed an impressive interfere in tumor progression.

### Combination of EZH2 and BET inhibitors epigenetically regulated several tumor-suppressors

**Fig. 5:**
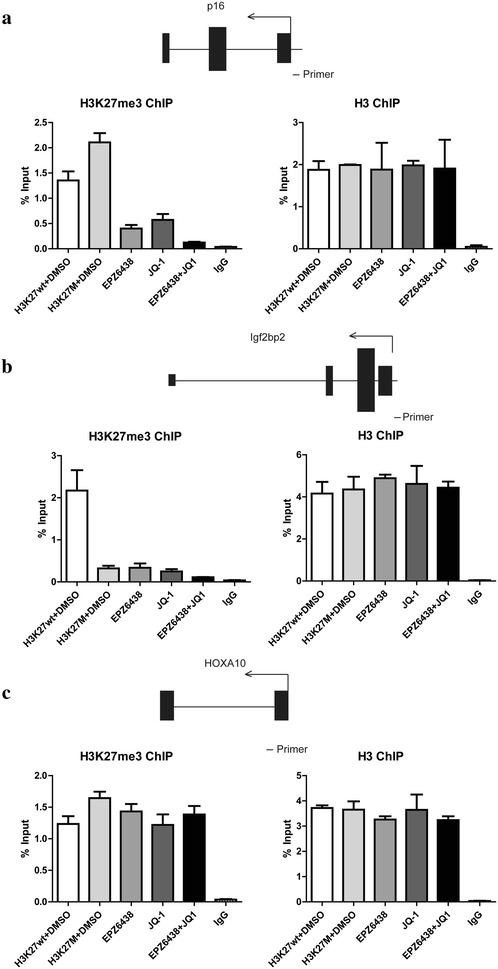
Combination of EZH2 and BET inhibitors epigenetically regulated tumor-surpressor like p16^Inka4^. ChIP-q-PCR analysis showing the enrichment of H3K27me3 (left panel) or H3 (right panel) as control over the p16^Ink4a^ gene (**a**), Igf2bp2 (**b**) and HOXA10 (**c**) in the NSCs with different treatment as indicated. The genes and the primer locations were presented on the top of the panel. Data were represented as mean ± SD; n = 3 independent experiments

**Fig. 6:**
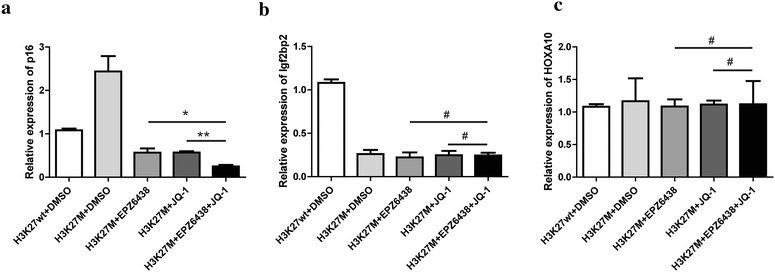
Gene expression level under the condition of combination of EZH2 and BET inhibitors. Q-PCR analysis showing the mRNA level of p16 (**a**), Igf2bp2 (**b**) and HOXA10 (**c**) gene with the different treatment as indicated. Data were represented as mean ± SD; n = 3 independent experiments. *p < 0.05, **p < 0.01 and ^#^p > 0.05

Several tumor-suppressors, including p16^INK4A^ and CDKN2A, have been reported to have the reduced expression due to the retain H3K27me3 activity in H3K27M induced DIPG tumors [16]. So we were interested whether combination of EPZ6438 and JQ-1 would also work on these epigenetic markers. We used ChIP-q-PCR to test the H3K27me3 levels on the p16^INK4A^ promoter. We could see the increased H3K27me3 on the p16^INK4A^ promoter and EPZ6438 or JQ-1 would dramatically reduce the H3K27me3 level while both of them showed totally abolish of the H3K27me3 activity (Fig. 5a). Igf2bp2 is a typical gene that will lose H3K27me3 with the expression of H3K27M, ChIP-q-PCR result showed that there is no effect of the inhibitors on this type of genes (Fig. 5b). H3K27M is reported to correlate with H3K27ac, however, this correlation is excluded by the PRC2 targets [29]. So we took the HOXA10 as an example to test the epigenetic changes in the PRC2 targets by the treatment of EPZ6438 and/or JQ-1. To our surprise, although the PRC2 targets also retained the H3K27me3 activity, neither of these two inhibitors would affect the H3K27me3 levels in the PDGFB/H3K27M NSCs (Fig. 5c). These results suggested that EPZ6438 and JQ-1 would change the epigenetic marks of the genes which been abnormally repressed in PDGFB/H3K27M NSCs, they would not affect the PRC2 targets to change their original repression pattern. Meanwhile, we detected the mRNA levels of these three different types of the genes, the expression levels were consistent with the ChIP results for these three types of the genes (Fig. 6).

### Combination of EZH2 and BET inhibitors improved survival in mice model

**Fig. 7:**
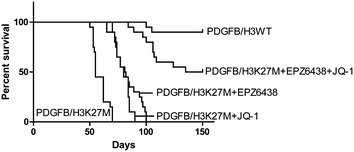
Combination of EZH2 and BET inhibitors improved survival in mice model. Survival curve of the mice injected into the pons with PDFGB/H3WT or PDGFB/H3K27M NSCs (1 × 10^5^). The inhibitor was given by intraperitoneal (ip) injection with the amount of EPZ6438 by 250 mg/kg and JQ-1 by 50 mg/kg. Each group has 20 mice

Finally, we tested the combination effect of EPZ6438 and JQ-1 on the mice model. The mice were implanted the PDGFB/H3K27M NSCs in the pons and treated with EPZ6438 and/or JQ-1 by tail vein injection. Treatment of the inhibitors individually showed the improvement in survival and the combination of these two inhibitors showed a prolonged survival which still had half of the mice alive after 150 days (Fig. 7).

## Discussion

DIPG is a highly aggressive pediatric brainstem tumor characterized by rapid and uniform patient demise. DIPGs usually grow quickly and affect important parts of the brain. The standard treatment for DIPG is radiation therapy, although it can dramatically improve patient’s condition, it usually recur after 6–9 months and progress rapidly. To find the new target and develop more specifically therapeutic approaches for DIPG became important to this tumor.

Previous studies uncovered that nearly 78% DIPG contained histone H3 gene mutations on lysine 27. Since human has dozens copies of Histones, H3K27M therefore constitutes a minor part (3.6–17.6%) of total Histone 3, this minor part of H3K27 mutation would make the global reduction of H3K27me3 levels [9]. However, recent work by ChIP-seq analysis showed that, several sets of genes retain H3K27me3 in H3K27M-mutant DIPGs [16, 27–29]. This is quite interesting cause some of these genes are tumor suppressors and the retaining of the H3K27me3 makes them keep silencing when the tumor under progress, which may be the potential target for the clinical treatment. Due to this, EZH2, which is the core member in PRC2 complex, was found to be required for the DIPG growth and it inhibitor had the effect on the tumor transplanted to the mice model. Meanwhile, when the nucleosomes lose H3K27me3 because of H3K27M occupation, they usually acquire H3K27ac, which results in the formation of H3K27M-K27ac heterotypic nucleosomes [9]. Piunti et al. found that bromodomain-containing protein 2 (BRD2) and BRD4 showed highly overlap with H3K27M-occupied sites [29]. This co-occupancy between bromodomain-containing protein and H3K27M-K27ac heterotypic nucleosomes indicates a potential role of BRD proteins in DIPG pathogenesis. Due to this, they used a well-known bromodomain and extra-terminal domain (BET) inhibitor, JQ-1, to treat the DIPG cells and animal model and demonstrated JQ-1 inhibited the tumor growth both in vitro and in vivo. Thus, BET inhibitors may also be a promising therapeutic strategy in DIPG.

Since DIPG is not a single gene disorder, develop more targets may be benefit in the clinical treatment. In this study, we test the combination effect of EZH2 inhibitor and BET inhibitor, in order to evaluate whether this combination is better for the treatment of DIPG. We generated H3K27M over-expressed mouse NSCs with the expression of PDGFB, which is proved to be similar to the human DIPG. Then we treated the cells with EPZ6438 and/or JQ-1. Our results showed that each inhibitor had the effect on the proliferation and apoptosis of the PDGFB/H3K27M NSCs, interestingly, combination of these two inhibitors exhibited better effect on suppressing growth of the tumor cells. In order to provide the detail mechanism, we examined the epigenetic markers of different kind of genes. p16^INK4A^ is a tumor-suppressor protein which acts as a cell-cycle inhibitor. Expression level of p16 will be strongly induced by stress and oncogene activation. ChIP-q-PCR showed p16 would has an increased H3K27me3 activity when H3K27M was expressed, this induction of H3K27me3 levels could be dramatically inhibited by EPZ6438 or JQ-1. Combination of these two inhibitors showed the better inhibition of H3K27me3 activity, which can activate the gene expression of p16 and thus to suppress the tumors. Igf2bp2 is another type of genes, which would lost H3K27me3 activity in the presence of H3K27M. This is the most cases because H3K27me3 is globally reduced when H3K27M exists in the cells. Under this condition, EPZ6438 or JQ-1 would not affect the H3K27me3 levels, since it is already lost. The last type of genes is the PRC2 targets. HOXA10 is a typical PRC2 target, which would retain its H3K27me3 levels even in the presence of H3K27M. This retaining of the H3K27me3 activity would make it keep silencing in the tumor cells. Interestingly, neither of the inhibitors of EZH2 and BET works on this type of genes. The results from SF8628 human DIPG cells revealed a genome-wide distribution of H3.3K27M that is highly correlated with active transcription, which were acetylated H3K27 (H3K27ac) and RNA polymerase II (RNA pol II) [29]. However, further study showed that H3K27M colocalizes with transcriptionally active chromatin regions were largely excluded from regions that are occupied by PRC2 and H3K27me3. In this case, PRC2 is almost entirely confined to chromatin regions marked with H3K27me3, and this mark is mutually exclusive with H3K27ac. If we compare the overlap between H3K27M and H3K27ac binding regions, it looks like H3K27M occupies many chromatin regions which EZH2 or PRC2 is unlikely [30]. That is the differences between the different loci in our Fig. 5 and maybe the reasons why cells transfected by H3K27M would be sensitive to EPZ6438 and JQ-1 in the loci like Fig. 5a. Thus, we demonstrated combination of EPZ6438 and JQ-1 would affected on the tumor suppressors which is abnormally repressed by H3K27M.

At last, we performed our results on the animal model, the mice transplanted with PDGFB/H3K27M NSCs were treated with EPZ6438 and/or JQ-1. Consistent with the in vitro data, the treatment of the inhibitors proved the survival of the mice and the combination showed the best effect. We realized it is better to generate DIPG xenograft model by primary human DIPG cell lines such as DIPG007, DIPG012, SF7761, SF8628, DIPG017 or DIPG018, unfortunately, we don’t have the way to get these cell lines. We also try to generate the cell lines for the human DIPG, but the specimen is limited to us. The xenograft model may provide the further information on the combination effect of these two inhibitors.

## Conclusions

In conclusion, in this study, we used combination of two inhibitors, one response on the inhibition of EZH2, another is for BET proteins. The combination of two inhibitors showed the better effect on the treatment of H3K27M DIPG cells by inhibiting the cell proliferation and promoting the apoptosis through epigenetic regulation of several tumor suppressor genes. These may provide EZH2 and BET proteins as the combined clinical therapy target for DIPG.

## Abbreviations

DIPG: diffuse intrinsic pontine glioma
PRCs: polycomb repressive complexes
NSCs: neural stem cells
